# Laterocervical Lymph Node Metastases in Papillary Thyroid Carcinoma: Predictive Factors for Recurrence and Oncological Outcome

**DOI:** 10.3390/jpm15100496

**Published:** 2025-10-16

**Authors:** Andrea Migliorelli, Marianna Manuelli, Agnese Maria Tringali, Claudio Moretti, Virginia Corazzi, Matteo Geminiani, Andrea Ciorba, Francesco Stomeo, Stefano Pelucchi, Chiara Bianchini

**Affiliations:** ENT & Audiology Unit, Department of Neurosciences, University Hospital of Ferrara, 44100 Ferrara, Italyvirginia.corazzi@unife.it (V.C.);

**Keywords:** lymph node ratio, papillary thyroid carcinoma, recurrence, thyroid N1b

## Abstract

**Background/Objectives**: Papillary cancer is the most common thyroid cancer. The development of lateral cervical lymph node metastases (I–V levels) is considered a major cause of recurrence. The aim of this study is to investigate the potential predictive factors for lateral cervical lymph node metastasis and disease recurrence, in order to tailor the clinical approach to these patients. An ROC (Receiver Operating Characteristic) curve has been set to search out a cut-off value for the lymph node ratio (LNR), a ratio of involved lymph nodes-to-examined lymph nodes, that could serve as an index of tumor recurrence. **Methods**: This was an observational retrospective study. The clinical charts of 43 patients with histopathological diagnosis of papillary thyroid cancer who underwent thyroidectomy with lateral and central neck dissection have been reviewed. These results have also been compared with those who underwent total thyroidectomy alone that served as a control group. **Results**: Extrathyroidal extension (*p*-value < 0.001), tumor size (*p*-value = 0.015), number of lymph nodes involvement (*p*-value = 0.022), and LNR (*p*-value = 0.004) were identified as potential predictors of tumor recurrence. The ROC curve revealed that an LNR value exceeding 0.205 is indicative of disease recurrence, with an Area Under the Curve (AUC) of 0.818, a sensitivity of 82%, and a specificity of 81%. Furthermore, fT4 value (*p*-value = 0.008), tumor size (*p*-value = 0.019), and alcohol consumption (*p*-value < 0.001) may serve as potential predictors of lymph node metastasis. **Conclusions**: Extrathyroidal extension, vascular invasion, tumor size, number of pathological lymph nodes, and LNR are associated with recurrence of papillary thyroid carcinoma; in particular, the lymph node ratio can be considered an effective indicator of recurrence risk.

## 1. Introduction

Thyroid cancer is one of the most prevalent cancers worldwide, with an incidence rate of 10.1 per 100,000 women and 3.1 per 100,000 men in the United States [1,2]. Recently, its incidence has been reported to increase, and it has been estimated to become the second most common cancer in women and the ninth in men by 2030 [3,4,5]. The etiology of thyroid cancer is not well understood. The only established risk factor is exposure to ionizing radiation, particularly during childhood. Recently, it has been suggested that genetics, increased body weight, exposure to hormones and certain environmental pollutants, working in the chemical industry, and living in polluted areas may be considered additional risk factors [1,3,4,5].

The histological classification of thyroid cancer is divided into three major subtypes: (i) differentiated carcinoma, (ii) medullary carcinoma, and (iii) anaplastic carcinoma. The most common histotype is papillary carcinoma, which is part of differentiated carcinomas, and accounts for approximately 90% of all thyroid cancers [6,7,8]. Papillary carcinoma has the best overall prognosis of all malignant histotypes, with an estimated 10-year specific survival rate of more than 96% [9,10]. Surgery is the treatment of choice, and is usually effective for disease management, possibly including neck dissection. The development of lymph node metastases in patients with papillary thyroid carcinoma is estimated at 30–80% [11]. While the prognosis for papillary carcinoma is favorable, with overall good survival, 3–10% of patients may experience a recurrence within the first decade after treatment [12,13]. Nowadays, it is crucial to perform accurate preoperative staging and to plan a personalized treatment and follow-up, as tailored as possible.

The primary staging systems currently employed are the eighth edition of the American Joint Committee on Cancer/Union for International Cancer Control (AJCC/UICC), the tumor-node-metastasis (TNM) staging system, and the American Thyroid Association (ATA) staging system. The former classifies patients with lymph node metastases of the central compartment as N1a and of the lateral compartment as N1b [14,15]. This classification is based purely on the topography of the lymph node pathology, without considering the number and size of the lymph nodes. In the seventh edition of the AJCC/UICC TNM classification, patients aged 45 years and older were divided into stage III or IVa, depending on whether they are N1a or N1b. In the eighth edition, this differentiation has been removed, considering them in a single stage [16]. The ATA proposed a risk stratification system categorizing the risk of recurrence as low, intermediate, or high, with the number of involved lymph nodes being a contributing factor [7].

The lymph node ratio (LNR) has been defined as the number of positive lymph nodes within the total number of lymph nodes removed. LNR has recently been used to assess the prognosis of other tumors, such as lung, gastric, colon, and head and neck squamous cell cancer [17,18,19,20]. LNR has been proposed in the literature as a possible prognostic factor also for papillary thyroid cancer patients with lymph node metastases [21]; in particular, most studies actually focus on the role of the LNR in level VI, or on the overall LNR without distinguishing between level VI and the levels in the lateral compartment.

Aiming to personalize the management of this disease, the purpose of this study was to investigate the potential predictive factors for lateral cervical lymph node metastasis and disease recurrence, particularly using a ROC curve to define a cut-off value for the LNR.

## 2. Materials and Methods

In this retrospective observational study, we have analyzed all patients who underwent total thyroidectomy with lateral neck dissection between 2003 and 2023 at the Department of Otolaryngology at the University Hospital of Ferrara.

Inclusion criteria for this study were as follows: (i) a definitive histological diagnosis of papillary thyroid carcinoma; (ii) neck dissection of levels between II and V with VI level. Therefore, patients with a definitive histological diagnosis other than papillary carcinoma (e.g., follicular, anaplastic, or medullary), patients who only performed level VI neck dissection, and patients with incomplete information on the number of lymph nodes examined and the number of lymph nodes involved were excluded from the study.

The charts of patients only treated by total thyroidectomy alone for papillary thyroid carcinoma have also been reviewed and served as control group.

A total of 147 charts of patients affected by papillary thyroid carcinoma, who underwent total thyroidectomy and lateral neck dissection at our hospital were retrieved; of these, only 43 have been included for this study, since having all the above data available for the analysis.

The following information have been retrieved, reviewing each chart: sex, age at the time of surgery, BMI (Body Mass Index), smoking history, alcohol consumption, family history of thyroid carcinoma, previous radiotherapy exposure, diabetes mellitus, hypertension, other malignancies in medical records, chronic obstructive pulmonary disease, surgical procedure performed, ipsilateral central compartment dissection, contralateral central compartment dissection, ipsilateral lateral compartment dissection, contralateral lateral compartment dissection, neck levels removed, days of hospitalization, complications, and preoperative laboratory values (thyroid-stimulating hormone [TSH], free triiodothyronine [fT3], free thyroxine [fT4], thyroglobulin, anti-thyroid peroxidase [TPO]). Regarding histopathological data, the following records were included: tumor histology focality, tumor size (considered as the largest diameter), resection margins (negative or positive), vascular invasion, lymphatic invasion, perineural invasion, extrathyroidal extension, concomitant thyroid inflammatory diseases, number of lymph nodes involved, and number of lymph nodes examined. Further information retrieved and included were as follows: lymph node ratio (the ratio of the number of lymph nodes involved to the number of lymph nodes examined), size of the largest lymph node, affected levels, extranodal extension, staging, possible adjuvant therapy (radioactive iodine [I-131] therapy, adjuvant radiotherapy, adjuvant chemotherapy, adjuvant immunotherapy, salvage surgery), follow-up time, recurrence, disease-free time, and overall survival.

This paper is an observational retrospective chart study, conducted in compliance with our institutional and national research ethical standards and in compliance with the Helsinki Declaration (2008). It was performed retrospectively through a systematic hospital case file review and therefore did not affect patient care in any way, since it only incorporates the recordings of a database and its evaluation.

### Statistical Analysys

A descriptive analysis of all variables with their frequencies was performed. The numerical data were expressed in absolute values, percentages, mean ± standard deviation, or median (range). Comparison between groups with regard to categorical variables was performed by means of the chi-square test or Fisher’s exact test in the case of expected frequencies < 5%. Student’s *t*-test was employed for analyzing continuous data when the variable data demonstrated a normal distribution.

A ROC curve was developed to determine the threshold value of lymph node ratio, indicative of tumor recurrence. Statistically significant results were defined as those with p-values less than 0.05. All analyses were performed using SPSS 29.0.1 software (IBM Corp., Armonk, NY, USA).

## 3. Results

Comparing the group with laterocervical neck dissection to the group without (control group of 43 patients), we noticed that patients with lymph node metastases underwent I-131 therapy more frequently (*p* < 0.001) and exhibited a higher recurrence rate (*p* = 0.007).

In addition, patients with lateral cervical lymph node metastases exhibited a prolonged hospitalization period (*p* < 0.001) in comparison to patients without metastases (control). Furthermore, patients with larger mean tumor sizes are more predisposed to developing lateral cervical metastases (*p* = 0.011) (Table 1).

Tumor size and fT4 values were found to be statistically significantly associated (*p* = 0.019 and *p* = 0.008, respectively) with the presence of laterocervical metastases (Table 2).

The demographic composition of the cohort is shown in Table 3. The patient population is composed of 28 women and 15 men, mean age of 54.20 years (±13.5). The analysis revealed that 18 (41.9%) patients had additional thyroid diseases, 33 (76.7%) patients had laterocervical lymph node metastases, and 11 (25.6%) patients developed recurrences (all recurrences occurred in areas of the neck that did not undergo dissection). In 13 (30.2%) patients, metastases of the lateral compartment alone were identified with no pathological lymph nodes in the central compartment. No patients underwent post-operative tracheotomy, salvage surgery, or adjuvant immunotherapy. The average follow-up time was 9.3 years (±5.4 years) and none of the patients died of thyroid disease.

Statistically significant associations were identified between recurrence of papillary thyroid carcinoma and contralateral neck dissection (*p* = 0.029), vascular invasion (*p* = 0.045), extrathyroidal tumor extension (*p* = 0.014), and the need for adjuvant radiotherapy (*p* = 0.011). Furthermore, patients who experienced a recurrence had a larger mean tumor size (*p* = 0.015), a larger number of involved lymph nodes (*p* = 0.022), and a higher lymph node ratio (*p* = 0.004) (Table 3).

A ROC curve was developed to determine the threshold value of the lymph node ratio (including only lymph nodes in the lateral compartment of the affected side), indicative of disease recurrence. This revealed an Area Under the Curve (AUC) of 0.82.

**Figure 1:**
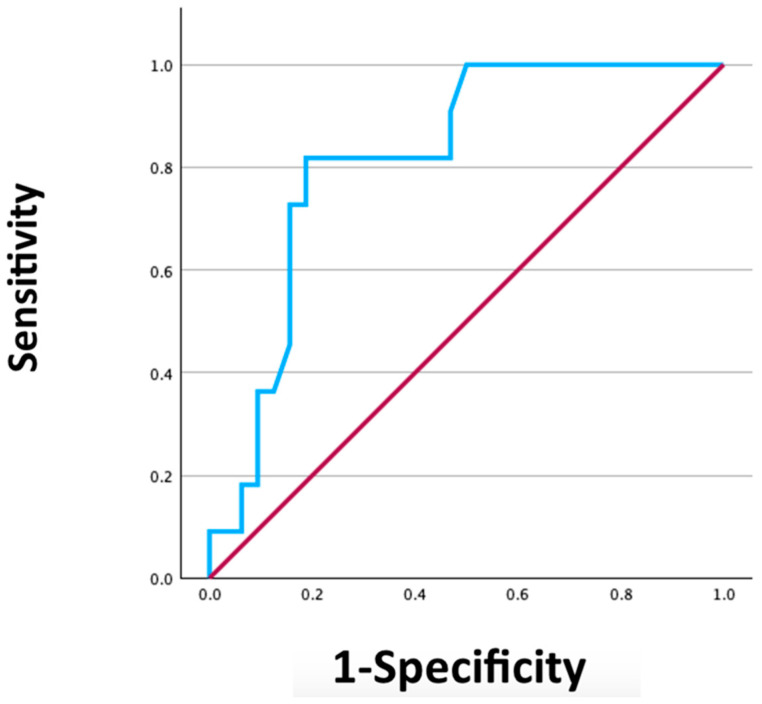
ROC curve of LNR threshold of recurrence.

This analysis identified a lymph node ratio cut-off value of 0.205, with a sensitivity of 82% and a specificity of 81% (Figure 1).

## 4. Discussion

The risk of lymph node metastasis within the lateral compartment can significantly impact the prognosis of patients diagnosed with papillary thyroid carcinoma. While the survival rate for this histotype is notably high (as no deaths attributable to the disease have been registered in the present cohort), the risk of recurrence represents the primary challenge, showing a significant effect on patients’ quality of life [22,23].

The findings of the present study demonstrate a correlation between tumor size and preoperative fT4 values with a high incidence of lateral lymph node compartment metastasis. The increase in fT4 could be related to an alteration in the TSH-fT4 axis, which can be linked to the presence of extensive disease. Furthermore, it has been observed that some metastases have the capacity to produce fT4 themselves, thus resulting in further increased circulation. Furthermore, it would be interesting to evaluate the role of exposure to environmental agents in the development of lateral cervical metastases from papillary carcinoma in the future. While some studies have shown that exposure to environmental factors may increase the risk of developing papillary thyroid carcinoma [1,3,4,5], there is currently no information on the role of these factors in the development of lateral cervical metastases.

The recurrence rate observed in the patient cohort undergoing thyroidectomy and lateral neck dissection was 25.6%. A multitude of factors have been identified to be related to the risk of recurrence, including vascular invasion, extrathyroidal extension, tumor size, the number of lymph nodes involved, and LNR.

The results of this study are consistent with those of the literature [24,25,26]. However, it should be noted that most of the available studies do not distinguish between lymph node metastases of the central and lateral neck compartment, which complicates the reliability of the results comparison. While papillary thyroid cancer typically metastasizes first to level VI and subsequently to the lateral neck compartment, some cases of skip metastasis have been documented, where the tumor metastasizes directly to the lateral levels [27]. Consequently, in our study, we have focused on a subgroup of patients who underwent lateral neck dissection, considering only the lymph nodes of the lateral compartment. The present study revealed the presence of skip metastases in approximately 30% of cases.

In addition, the most widely employed classifications (ATA and AJCC/UICC) are affected by some limitations, frequently complicating the stratification of patients with N1b papillary carcinoma, as per the existing criteria [7,14,15,16]. Consequently, the LNR has recently been introduced also in the staging of thyroid cancer, representing a promising parameter in assessing the risk of disease recurrence [21]. This feature could represent a promising tool in the era of precision medicine in order to provide a tailored management of this disorder. LNR evaluates two factors, as follows: (i) the number of positive lymph nodes, which reflects the regional spread of the disease; and (ii) the neck surgery radicality, represented by the total number of excised lymph nodes. The LNR value provides a relative parameter that is more predisposed to general applicability, rather than a single absolute parameter that may be influenced by pathological sampling or surgical technical inadequacies [20]. To date, LNR has been predominantly correlated with survival rate. Schneider et al. analyzed 10,955 cases and found that an LNR value ≥ 0.42 was associated with disease-specific mortality, similar results also found by Kim et al. with a lateral LNR cut-off greater than 0.3 [28,29]. Furthermore, patients exhibiting a higher overall LNR have a higher risk of incomplete response to therapy and thus a higher risk of recurrence [30]. To the best of our knowledge, only a few studies have evaluated the value of lateral LNR alone in predicting the risk of disease recurrence. The findings of the present study indicate that a cut-off of 0.205 demonstrates satisfactory sensitivity and specificity levels of 81% and 82%, respectively, in predicting the risk of recurrence, with an Area Under the Curve (AUC) value of 0.818. To date, the cut-off value of the LNR has been examined in a limited number of studies. Yuksel et al. reported that a cut-off of 0.21 for LNR was a predictor of disease-free survival in N1b patients, Lee et al. found a cut-off of 0.218, Park et al. of 0.22, and Kang et al. of 0.23 [24,31,32,33]. Therefore, a cut-off value between 0.20 and 0.23 appears to be associated with a higher probability of recurrence in N1b patients. Consequently, patients diagnosed with papillary thyroid cancer and exhibiting an LNR greater than 0.20 should be considered for adjuvant therapies and subjected to more rigorous post-operative follow-up measures, as per their higher risk of post-operative recurrence.

The integration of LNR with TNM classification systems has been demonstrated in the existing literature to enhance its predictive capacity for the evaluation of recurrences. Also, the combination of ATA or the AJCC/UICC eighth edition risk stratification plus LNR could improve the stratification of the recurrence risk [34,35]. Therefore, in our opinion, this parameter should be promptly incorporated into the revised papillary thyroid cancer guidelines, to better assess the risk of recurrence in these patients, also allowing personalized treatment and follow-up.

Major drawbacks of this study are the following: (i) the small number of patients included; (ii) the single-institution experience; (iii) the retrospective nature of the study; and (iv) the lack of an external validation cohort.

## 5. Conclusions

According to the results of the present study, when considering only lymph nodes of the lateral neck compartment, a LNR cut-off value of 0.205 has a good sensitivity and specificity in identifying patients at risk of recurrence for papillary thyroid carcinoma.

Furthermore, several other parameters such as extrathyroidal tumor extension, vascular invasion, tumor size, number of pathological lymph nodes, and LNR have been associated with disease recurrence in the present series.

In our opinion, enhancing the knowledge of predictive factors in papillary thyroid cancer, particularly improving the stratification of the recurrence risk, could allow safer and more accurate management of these patients. Further prospective and multicenter studies with larger case series are necessary to confirm the results of this study.

## Figures and Tables

**Table 1:** Comparison of the studied population vs. control group.

		Total Thyroidectomy and Neck Dissection (n = 43)	Total Thyroidectomy (n = 43)	*p*-Value
Gender (%)	Female	28 (32.6%)	24 (27.9%)	0.378
	Male	15 (17.4%)	19 (22.1%)	
Average Age at Surgery (±SD)		54.8 (±13.5)	52.3 (±16.1)	0.218
Smoking History (%)	Yes	17 (20.2%)	10 (11.9%)	0.102
	No	25 (29.8%)	32 (38.1%)	
Average BMI (±SD)		27.6 (±5.9)	27.7 (±5.8)	0.197
Average TSH (±SD) µIU/mL		2.4 (±1.7)	2.7 (±1.9)	0.337
Average fT3 (±SD) pg/mL		3.5 (±1.1)	3.4 (±1.2)	0.459
Average fT4 (±SD) pg/mL		15.5 (±8.8)	16.7 (3.2)	0.269
Average Days of Hospitalization (±SD)		5.7 (±3.9)	2.9 (±0.6)	<0.001
Tumor Focality (%)	Multifocal	26 (30.6%)	20 (23.5%)	0.154
	Unifocal	16 (18.8%)	23 (27.1%)	
Average Tumor Size (±SD) cm		14.9 (±9.6)	10.8 (±6.2)	0.011
Margins (%)	Positive	23 (26.7%)	21 (24.4%)	0.666
	Negative	20 (23.3%)	22 (25.6%)	
Vascular Invasion (%)	Yes	4 (4.7%)	1 (1.2%)	0.360
	No	39 (45.3%)	42 (48.8%)	
Lymphatic Invasion (%)	Yes	2 (2.3%)	0 (0%)	0.494
	No	41 (47.7%)	43 (50%)	
Extrathyroid Extension (%)	Yes	17 (19.8%)	11 (12.8%)	0.167
	No	26 (30.2%)	32 (37.2%)	
I-131 (%)	Yes	40 (46.5%)	27 (31.4%)	<0.001
	No	3 (3.5%)	16 (18.6%)	
Adjuvant Radiotherapy (%)	Yes	5 (5.8%)	0 (0%)	0.055
	No	38 (44.2%)	43 (50%)	
Recurrence (%)	Yes	11 (12.8%)	2 (2.3%)	0.007
	No	32 (37.2%)	41 (47.7%)	

Abbreviation legend. SD: Standard Deviation; BMI: Body Max Index; TSH: thyroid-stimulating hormone; fT3: free triiodothyronine; fT4: free thyroxine; I-131: radioactive iodine therapy.

**Table 2:** Association of clinical-pathological features and lateral neck lymph node metastasis.

		Positive Lymph Node	Negative Lymph Node	*p*-Value
Gender (%)	Female	19 (44.2%)	9 (20.9%)	0.127
	Male	14 (32.6%)	1 (2.3%)	
Average Age at Surgery (±SD)		53.9 (±14.1)	57.7 (±11.5)	0.204
Smoking History (%)	Yes	15 (35.7%)	2 (4.8%)	0.162
	No	17 (40.5%)	8 (19%)	
Average BMI (±SD)		26.7 (±5.9)	27.9 (±6)	0.295
Average TSH (±SD) µIU/mL		2.4 (±1.8)	2.4 (±1.6)	0.484
Average fT3 (±SD) pg/mL		3.6 (±1.3)	3.1 (±0.3)	0.155
Average fT4 (±SD) pg/mL		16.4 (±9.5)	11.6 (±0.2)	0.008
Thyroglobulin (%)	>5000 ng/mL	10 (38.5%)	1 (3.8%)	0.614
	<5000 ng/mL	12 (46.2%)	3 (11.5%)	
Tumor Focality (%)	Multifocal	22 (52.4%)	4 (9.5%)	0.142
	Unifocal	10 (23.8%)	6 (14.3%)	
Average Tumor Size (±SD) cm		16.1 (±10.4)	11(±4.7)	0.019

Abbreviation legend. SD: Standard Deviation; BMI: Body Max Index; TSH: thyroid-stimulating hormone; fT3: free triiodothyronine; fT4: free thyroxine.

**Table 3:** Clinical-pathological features of the studied population and association with recurrence.

		Recurrence (n = 11)	Not Recurrence (n = 32)	Total (n = 43)	*p*-Value
Gender (%)	Female	7 (16.3%)	21 (48.8%)	28 (65.1%)	1
	Male	4 (9.3%)	11 (25.6%)	15 (34.9%)	
Average Age at Surgery (±SD)		52.91 (±17.8)	55.50 (±11.9)	54.20 years (±13.5)	0.330
Smoking History (%)	Yes	5 (11.9%)	12 (28.6%)	17 (40.5%)	0.733
	No	6 (14.3%)	19 (45.2%)	25 (59.5%)	
Average BMI (±SD)		28.9 (±5.9)	27.2 (±5.9)	27.6 (±5.9)	0.198
Thyroid Cancer Family History (%)	Yes	2 (4.8%)	2 (4.8%)	4 (9.5%)	0.277
	No	9 (21.4%)	29 (69%)	38 (90.5%)	
Presence of Level VI Lymph Nodes (%)	Yes	10 (23.8%)	23 (54.8%)	33 (78.6%)	0.403
	No	1 (2.4%)	8 (19%)	9 (21.4%)	
Presence of Controlateral level II-V Lymph Nodes (%)	Yes	4 (9.3%)	2 (4.7%)	6 (14%)	0.029
	No	7 (16.3%)	30 (69.7%)	37 (86%)	
Average TSH (±SD) µIU/mL		2.7 (±1.9)	2.3 (±1.7)	2.4 (±1.7)	0.307
Average fT3 (±SD) pg/mL		3.6 (±0.9)	3.4 (±1.6)	3.5 (±1.1)	0.400
Average fT4 (±SD) pg/mL		14.7 (±4.6)	15.7 (±9.8)	15.5 (±8.8)	0.356
Average Anti-TPO (±SD) IU/mL		59 (±95.9)	67.9 (±121.4)	65.8 (±113.1)	0.442
Thyroglobulin (%)	>5000 ng/mL	3 (11.5%)	8 (30.8%)	11 (42.3%)	1
	<5000 ng/mL	3 (11.5%)	12 (46.2%)	15 (57.7%)	
Average Days of Hospitalization (±SD)		7.9 (±5.5)	5 (±2.9)	5.7 (±3.9)	0.060
Tumor Focality (%)	Multifocal	8 (19%)	18 (42.9%)	26 (61.9%)	0.270
	Unifocal	2 (4.8%)	14 (33.3%)	16 (38.1%)	
Average Tumor Size (±DS) cm		22.1 (±11.5)	12.6 (±7.7)	14.9 (±9.6)	0.015
Margins (%)	Positive	8 (18.6%)	15 (34.9%)	23 (53.5%)	0.138
	Negative	3 (7%)	17 (39.5%)	20 (46.5%)	
Vascular Invasion (%)	Yes	3 (7%)	1 (2.3%)	4 (9.3%)	0.045
	No	8 (18.6%)	31 (72.1%)	39 (90.7%)	
Lymphatic Invasion (%)	Yes	1 (2.3%)	1 (2.3%)	2 (4.7%)	0.451
	No	10 (23.3%)	31 (72.1%)	41 (95.3%)	
Perineural Invasion (%)	Yes	0 (0%)	1 (2.3%)	1 (2.3%)	1
	No	11 (25.6%)	31 (72.1%)	42 (97.7%)	
Extrathyroid Extension (%)	Yes	8 (18.6%)	9 (20.9%)	17 (39.5%)	0.014
	No	3 (7%)	23 (53.5%)	26 (60.5%)	
Concomitant Thyroid Disorders (%)	Yes	4 (9.3%)	14 (32.6%)	18 (41.9%)	0.736
	No	7 (16.3%)	18 (41.8%)	25 (58.1%)	
Average Number Lymph Node Involved (±SD) (II–V affected side)		14.1 (±15.9)	2.9 (±4.2)	5.8 (±9.9)	0.022
Average LNR (±SD) (II–V affected side)		0.35 (±0.21)	0.14 (±0.18)	0.19 (±0.20)	0.004
Average Largest Lymph Node Size (±SD) (II–V affected side)		34.3 (±25.8)	20.4 (±11.6)	25.2 (±18.2)	0.060
Extranodal Extension (%)	Yes	1 (2.4%)	1 (2.4%)	2 (4.8%)	0.424
	No	9 (21.4%)	31 (73.8%)	40 (95.2%)	
I-131 (%)	Yes	10 (23.3%)	30 (69.7%)	40 (93%)	1
	No	1 (2.3%)	2 (4.7%)	3 (7%)	
Adjuvant Radiotherapy (%)	Yes	4 (9.3%)	1 (2.3%)	5 (11.6%)	0.011
	No	7 (16.3%)	31 (72.1%)	38 (88.4%)	
Adjuvant Chemotherapy (%)	Si	1 (2.3%)	0 (0%)	1 (2.3%)	0.256
	No	10 (23.3%)	32 (74.4%)	42 (97.7%)	

Abbreviation legend. SD: Standard Deviation; BMI: Body Max Index; TSH: thyroid-stimulating hormone; fT3: free triiodothyronine; fT4: free thyroxine; anti-TPO: anti-thyroid peroxidase; LNR: lymph node ratio; I-131: radioactive iodine therapy.

## Data Availability

The original contributions presented in this study are included in the article; further inquiries can be directed to the corresponding author.
